# Peroxisomal polyamine oxidase and NADPH-oxidase cross-talk for ROS homeostasis which affects respiration rate in *Arabidopsis thaliana*

**DOI:** 10.3389/fpls.2014.00132

**Published:** 2014-04-03

**Authors:** Efthimios A. Andronis, Panagiotis N. Moschou, Imene Toumi, Kalliopi A. Roubelakis-Angelakis

**Affiliations:** ^1^Laboratory of Plant Physiology and Biotechnology, Department of Biology, University of CreteHeraklion, Greece; ^2^Department of Plant Biology, Uppsala BioCenter, Swedish University of Agricultural Sciences and Linnean Center for Plant BiologyUppsala, Sweden

**Keywords:** polyamines, NADPH-oxidase, polyamine oxidases, respiration, ROS homeostasis, *Arabidopsis*

## Abstract

Homeostasis of reactive oxygen species (ROS) in the intracellular compartments is of critical importance as ROS have been linked with nearly all cellular processes and more importantly with diseases and aging. PAs are nitrogenous molecules with an evolutionary conserved role in the regulation of metabolic and energetic status of cells. Recent evidence also suggests that polyamines (PA) are major regulators of ROS homeostasis. In *Arabidopsis* the backconversion of the PAs spermidine (Spd) and spermine to putrescine and Spd, respectively, is catalyzed by two peroxisomal PA oxidases (AtPAO). However, the physiological role of this pathway remains largely elusive. Here we explore the role of peroxisomal PA backconversion and in particular that catalyzed by the highly expressed AtPAO3 in the regulation of ROS homeostasis and mitochondrial respiratory burst. Exogenous PAs exert an NADPH-oxidase dependent stimulation of oxygen consumption, with Spd exerting the strongest effect. This increase is attenuated by treatment with the NADPH-oxidase blocker diphenyleneiodonium iodide (DPI). Loss-of-function of *AtPAO3* gene results to increased NADPH-oxidase-dependent production of superoxide anions ($O_{2}^{•-}$ ), but not H_2_O_2_, which activate the mitochondrial alternative oxidase pathway (AOX). On the contrary, overexpression of *AtPAO3* results to an increased but balanced production of both H_2_O_2_ and $O_{2}^{•-}$ . These results suggest that the ratio of $O_{2}^{•-}$ /H_2_O_2_ regulates respiratory chain in mitochondria, with PA-dependent production of $O_{2}^{•-}$ by NADPH-oxidase tilting the balance of electron transfer chain in favor of the AOX pathway. In addition, AtPAO3 seems to be an important component in the regulating module of ROS homeostasis, while a conserved role for PA backconversion and ROS across kingdoms is discussed.

## INTRODUCTION

Polyamines (PA) are low-molecular mass nitrogenous compounds, and the most abundant ones, across kingdoms, are putrescine (Put), spermidine (Spd), spermine (Spm), and thermospermine (t-Spm); they have been correlated with plethora of biological processes, including protein regulation (Baron and Stasolla, 2008; Takahashi and Kakehi, 2010), development (Wimalasekera et al., 2011; *inter alia*), ion channels (Wu et al., 2010; Zepeda-Jazo et al., 2011), control of nitrogen: carbon balance (Mattoo et al., 2006; for review see Moschou et al., 2012), stress responses (Alcazar et al., 2011b; Marco et al., 2011; Moschou and Roubelakis-Angelakis, 2013) and in particular homeostasis of reactive oxygen species (ROS; Chattopadhyay et al., 2006; *inter alia*).

Polyamines catabolism is mediated mainly by two classes of amine oxidases (AO), the diamine oxidases (DAO) and the PA oxidases (PAO; reviewed in Moschou et al., 2012). In *Arabidopsis*, the AO pathway consists of several, perhaps functionally redundant genes. For example, *Arabidopsis* has at least 10 *DAO* genes (four have been characterized; Møller and McPherson 1998; Planas-Portell et al., 2013) and five *PAO* genes (*AtPAO1–AtPAO5*, all have been characterized; Ahou et al., 2014). DAOs oxidize Put and cadaverine (Cad), and with much lower affinity, Spd and Spm. The action of DAOs on Put yields pyrroline, H_2_O_2_, and ammonia (NH^4^^+^; Cohen, 1998).

In contrast to DAOs, PAOs oxidize Spd, and Spm but not Put (Angelini et al., 2010). The apoplastic PAO catalyzes the terminal oxidation of PAs, yielding pyrroline and 1-(3-aminopropyl) pyrrollinium from Spd and Spm, respectively, along with 1,3-diaminopropane and H_2_O_2_. The plant intracellular (cytoplasmic or peroxisomal) PAOs interconvert PAs, producing H_2_O_2_. Interestingly, they interconvert Spm to Spd and Spd to Put, reversing the PA biosynthetic pathway (Tavladoraki et al., 2006; Kamada-Nobusada et al., 2008; Moschou et al., 2008c; Toumi et al., 2010; Fincato et al., 2012).

Polyamines catabolism has been correlated with numerous processes including cell growth, development, stress responses, and programmed cell death (PCD; Møller and McPherson, 1998; Yoda et al., 2003, 2006; Paschalidis and Roubelakis-Angelakis, 2005a, b; Tisi et al., 2011; Moschou et al., 2012; Moschou and Roubelakis-Angelakis, 2013). We have documented the contribution of tobacco apoplastic PAO (Moschou et al., 2008b) and peroxisomal (Wu et al., 2010) AtPAO3 in H_2_O_2_ production. The apoplastic pathway has been mostly correlated with the execution of PCD (Yoda et al., 2003, 2006; Moschou et al., 2008b; Fincato et al., 2012; Moschou and Roubelakis-Angelakis, 2013). The peroxisomal AtPAO3 is critical for the elongation of pollen tube by modulating a plasma membrane H_2_O_2_-dependent Ca^2^^+^-influx channel (Wu et al., 2010). In *Arabidopsis*, PA oxidation is mediated by PAO with diverse specificities and expression patterns (Fincato et al., 2011), thereby regulating ROS levels in a complex manner.

Superoxides ($O_{2}^{•-}$ ) and H_2_O_2_ are the most well studied ROS; they are important players in physiological and pathological processes (Pitzschke et al., 2006; Dikalov et al., 2011; Suzuki et al., 2013). NADPH-oxidase catalyzes the conversion of molecular oxygen to $O_{2}^{•-}$ , and its activation accounts mostly for the large consumption of oxygen that characterizes the respiratory burst in mammalian phagocytic cells (Vignais, 2002). In mammals NADPH-oxidase is composed of membrane-bound and cytosolic proteins. In the center of the NADPH oxidase complex lies the heterodimeric NADPH-binding flavocytochrome b558, consisting of the glycosylated transmembrane protein gp91phox and the non-glycosylated p22phox subunit. Upon activation, the cytosolic proteins p47phox and p67phox become phosphorylated and translocate, together with p40phox and p21rac, to the membrane components, to form the active NADPH-oxidase complex (Segal and Abo, 1993). Plants deficient in gp91phox homologs have compromised responses to stress and have a reduced ability to accumulate ROS. Antisense tomato lines (*Lerboh1*) show reduced ROS accumulation and compromise wound response (Sagi et al., 2004). *Arabidopsis* plants disrupted in the *gp91phox* homologs, the respiratory burst oxidase homolog D (*AtrbohD*) and *AtrbohF*, exhibit reduced ROS production and treatment with the avirulent bacterium *Pseudomonas syringae* pv tomato DC3000 results to cell death (Torres et al., 2005), whereas they have diminished stomatal closure in response to abscissic acid (ABA; Kwak et al., 2003). These data suggest that NADPH-oxidase homologs in plants are important for ROS accumulation.

Another important source of ROS is the mitochondrial electron transport chain (ETC; Muller et al., 2004; Vacca et al., 2004). It consists of four complexes, tightly bound to the intermembrane space of mitochondria. Electrons derived from the tricarboxylic acid (TCA) cycle in the matrix move toward the ETC and in turn pass through the four complexes. Transfer of electrons between complex 3 and 4 of the ETC is facilitated *via* the electron carrier cytochrome-c (cyt-c pathway). The electron motion generates a proton gradient which in turn drives an ATPase. Dysfunction of the mitochondrial ETC leads to the leakage of electrons toward oxygen resulting in the generation of $O_{2}^{•-}$ (Muller et al., 2004). In order to dissipate the excess electrons, the mitochondria possess another pathway, the alternative pathway, which depends on an alternative terminal oxidase (AOX; Atkin et al., 2002). AOX alleviates mitochondrial ETC from the excess electron load (Yip and Vanlerberghe, 2001).

Previous work from our lab suggested that a regulatory crosstalk between PAs and NADPH-oxidase takes place during tobacco protoplast regeneration (Papadakis and Roubelakis-Angelakis, 2005). PAs seem to be necessary for protoplasts to retain their totipotent state, and prevention of PCD. The interaction between main cellular sources of ROS, such as mitochondria and NADPH-oxidases, however, remains obscure. More importantly, a feed-forward regulation of different ROS sources has been reported (Dikalov et al., 2011). Therefore, the regulatory crosstalk between ROS sources merits further examination.

Here, we report that exogenous PAs stimulate oxygen consumption in *Arabidopsis* in an NADPH-oxidase dependent manner. Plants overexpressing the peroxisomal AtPAO3 show decreased oxygen consumption rate, in strict contrast to loss-of-function *Atpao3* plants which show increased consumption through the AOX pathway. Surprisingly, this increase is attenuated by diphenyleneiodonium iodide (DPI) but not by ascorbate (ASA), suggesting that NADPH-oxidase is upstream of a respiratory increase mediated by AOX. By delving the regulatory function of $O_{2}^{•-}$ in oxygen consumption rate, we found that *AtPAO3* overexpressing plants show a balanced production of both $O_{2}^{•-}$ and H_2_O_2_, while *Atpao3* loss-of-function plants show a high ratio of $O_{2}^{•-}$ *versus* H_2_O_2_ production. These data suggest that NADPH-oxidase and AtPAO3 cross-talk for balancing intracellular $O_{2}^{•-}$/H_2_O_2_ which in turn affect the cyt-c/AOX pathways.

## MATERIALS AND METHODS

### PLANT MATERIAL AND GROWTH CONDITIONS

*Arabidopsis thaliana* wild type (WT) plants of the ecotype Columbia (Col-0) were used along with transgenic plants overexpressing the peroxisomal *AtPAO3* (S-*AtPAO3*) and *Atpao3* T-DNA loss-of-function insertional mutants, previously described (Moschou et al., 2008c; Wu et al., 2010; Fincato et al., 2011). Plants were grown in a cabinet using an 8/16 h (light/dark) photoperiod and a constant temperature of 23°C. Developing seedlings were transferred to 96-well plates filled with 1/4 strength Murashige and Skoog (MS; Murashige and Skoog, 1962) culture medium. All treatments were carried out by supplementing the culture medium with the corresponding agent. More specifically, the PAs Put, Spd, and Spm were added as aqueous solutions at a final concentration of 1mM. Control plants were mock treated with dH_2_O.

### POLAROGRAPHIC MEASUREMENT OF RESPIRATORY OXYGEN CONSUMPTION

The rate of oxygen consumption was essentially determined as previously described (Andronis and Roubelakis-Angelakis, 2010). In brief, polarography was performed at 30°C with a Clark type electrode system (Hansatech Instruments, Kings’s Lynn, Norfolk, UK), in the presence and absence of the alternative respiratory inhibitor salicylhydroxamic acid (SHAM). Oxygen consumption was measured for a period of 5 min. For inhibitor treatments, leaves were incubated in 15 mM SHAM in 3% (v/v) methanol for a period of 10 min prior to measurement. Control leaves were incubated in dH_2_O or 3% (v/v) methanol. In all cases, the rate of oxygen consumption was expressed as per g fresh weight.

### NADPH OXIDASE NATIVE PAGE AND ACTIVITY STAINING

Separation of NADPH oxidase isoenzymes and activity staining were carried out according to Carter et al. (2007). *Arabidopsis* leaf tissue was collected and ground using liquid N_2_. The powder was homogenized in a buffer containing 50 mM sodium phosphate, pH 6.8, supplemented with 0.5% (v/v) Triton X-100. 100 μg of protein were separated using native PAGE at 40 mA. Gels were then incubated in 0.5 mg mL^-1^ nitroblue tetrazolium (NBT) in 10 mM Tris, pH 7.4, and 134 mM NADPH until bands were detected.

### IN SITU DETECTION OF H_2_O_2_ and $O_{2}^{•-}$

*In situ* accumulation of H_2_O_2_ was detected using the method of Thordal-Christensen et al. (1997) and of $O_{2}^{•-}$ according to Jabs et al. (1996). *Arabidopsis* seedlings were destained using boiling pure ethanol and photographed using a Nikon Coolpix 4500 digital camera.

### PROTEIN GEL BLOT ANALYSES AND IN-GEL ACTIVITIES OF APX AND SOD

Protein extractions and gel blots were performed as previously described (Moschou et al., 2013). One hundred mg of leaf material was mixed with 100 μL of urea extraction buffer [4 M urea, 100 mM DTT, and 1% (v/v) Triton X-100] and incubated in ice for 10 min. The samples were boiled with Laemmli sample buffer for 10 min and centrifuged at 13,000 rpm for 15 min. Equal amounts of the supernatants were loaded on 10% (v/v) polyacrylamide gels and blotted on a polyvinylidene difluoride (PVDF) membrane.

For the activity staining of ascorbate peroxidase (APX), 10 mM ASA were added to isoelectric focusing electrophoresis buffer (Rao et al., 1995), and 10% gels were pre-run for 30 min at 20 mA. Subsequently, the gels were incubated in the dark in a solution containing 50 mM potassium phosphate buffer, pH 7.0, and 2 mM ASA; the gels were incubated in the dark for another 30 min in 50 mM potassium phosphate buffer, pH 7.0, 4 mM ASA, and 2 mM H_2_O_2_. Bands were visualized after the incubation of gels in coloring solution (50 mM potassium phosphate buffer, pH 7.8, 14 mM tetramethylethylenediamine, TEMED; and 1.2 mM NBT). The activity staining of superoxide dismutase (SOD) has been previously described (Beauchamp and Fridovich, 1971).

### IMAGE AND STATISTICAL ANALYSIS

The image analysis was performed using ImageJ v 1.41 software^1^. Statistical analysis was performed with SPSS v14^2^ or JMP v 9 software^3^. We used Dunett’s test with alpha values set at α = 0.1.

## RESULTS

### EXOGENOUS PAs STIMULATE OXYGEN CONSUMPTION RATE

Our previous work established the effect of abiotic stress on the respiratory activity of WT tobacco plants (Andronis and Roubelakis-Angelakis, 2010). Under abiotic stress conditions, cyt-c, an electron carrier located between complexes III and IV of the ETC, dissociates leading to malfunction of the mitochondrial ETC and accumulation of ROS. As a result, the AOX pathway is activated in order to dissipate the excess electrons “leaking” from the ETC. Taking into consideration the link between PAs and plant responses to stresses, we attempted to reveal a potential correlation between PAs and respiratory activity in *Arabidopsis thaliana*.

**FIGURE 1:**
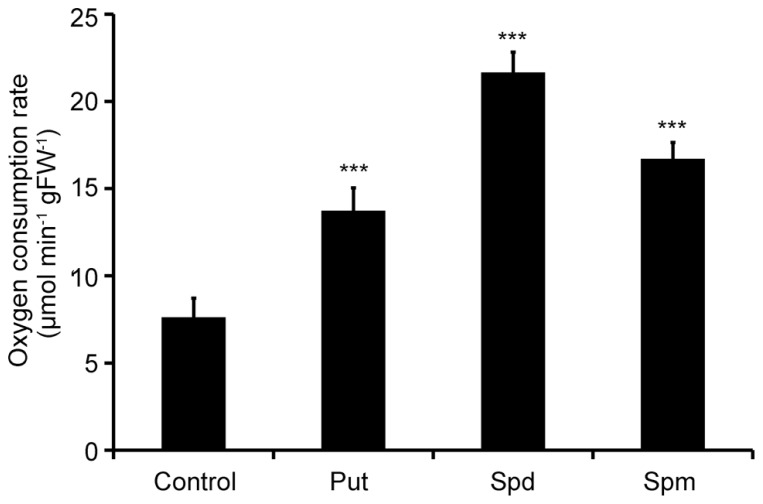
**Effect of exogenous polyamines on oxygen consumption rate in *Arabidopsis*.** *Arabidopsis* Col-0 seedlings were treated with 1 mM Put, Spd, or Spm for a period of 10 min, and oxygen consumption rate was estimated by a Clark-type electrode. Data are the means of three independent experiments ±SD. Asterisks indicate statistical significant differences (***P < 0.001).

Two-week old Col-0 *Arabidopsis* seedlings were treated with exogenous Put, Spd, and Spm and oxygen consumption rate was determined 10 min post-treatment. Respiration rate increased in the presence of all PAs used, in terms of oxygen consumption. Spd exerted the strongest effect on the respiration rate, resulting in a 2.6-fold increase compared to the mock treated plants (**Figure 1**). Put and Spm increased respiration, by 1.8- and 2.1-fold, respectively, compared to mock treated plants. These findings revealed an apparent link between PAs and ETC regulation in plants.

### STIMULATION OF OXYGEN CONSUMPTION RATE DEPENDS ON PA- INDUCED GENERATION OF $O_{2}^{•-}$

**FIGURE 2:**
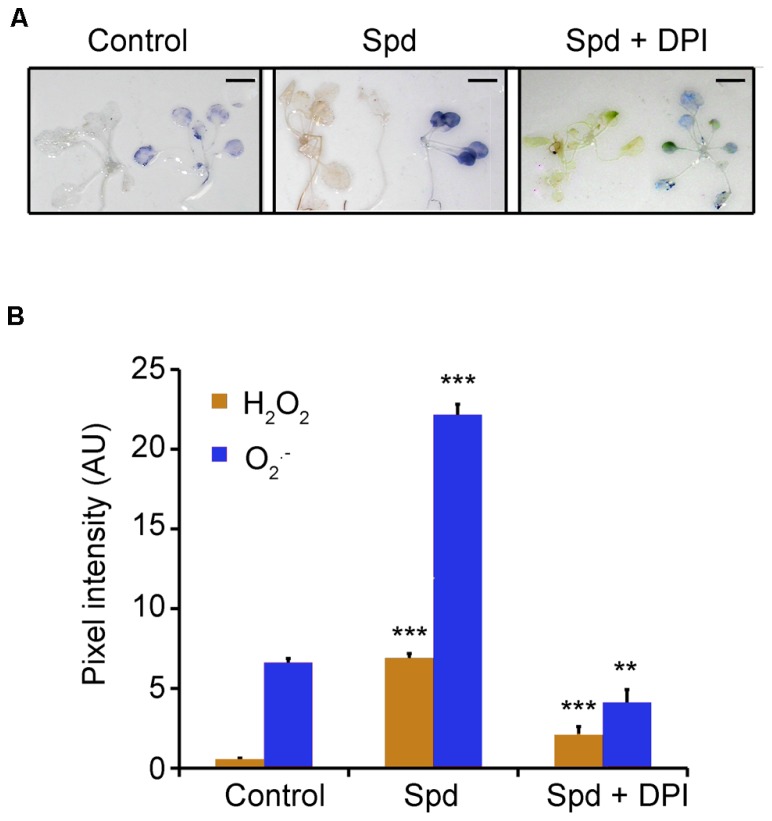
**Effect of exogenous Spd and DPI on ROS homeostasis in *Arabidopsis* plants. (A)** Plants were treated with 1 mM Spd for a period of 10 min and H_2_O_2_ or $O_{2}^{•-}$ were estimated by *in situ* detection methods. Scale bars, 2.8 cm. **(B)** Relative pixel intensity of the brownish (detection of H_2_O_2_) or bluish (detection of $O_{2}^{•-}$ ) adduct formed after application of 1 mM Spd. AU, arbitrary units. Data are the means of three independent experiments ±SD. Asterisks indicate statistical significant differences (***P < 0.001; **P < 0.01).

Previously, we have shown that exogenous application of Spd to tobacco plants leads to a significant increase in H_2_O_2_ content generated by the action of PAO (Moschou et al., 2008b). A plausible hypothesis could be that PAO-dependent ROS production is a component of the pathway which is responsible for the observed increase of the oxygen consumption rate. To test this hypothesis, we firstly examined whether exogenous Spd could induce H_2_O_2_ and $O_{2}^{•-}$ accumulation in *Arabidopsis*. To this end, we employed *in situ* detection protocols of H_2_O_2_ and $O_{2}^{•-}$ . Indeed, a 10 min treatment with exogenous Spd (1 mM) led to a significant accumulation of H_2_O_2_, as a result of PA oxidation. The use of 1 mM Spd was based on our previous findings that this concentration is enough to enter peroxisomes and be backconverted to Put in *Arabidopsis* (Fincato et al., 2011). Surprisingly, we found a significant increase in $O_{2}^{•-}$ content, as well (**Figures 2A,B**). From the aforementioned it is evident that the increase in plant respiration coincided with elevated H_2_O_2_ and/or $O_{2}^{•-}$ in treated plants.

**FIGURE 3:**
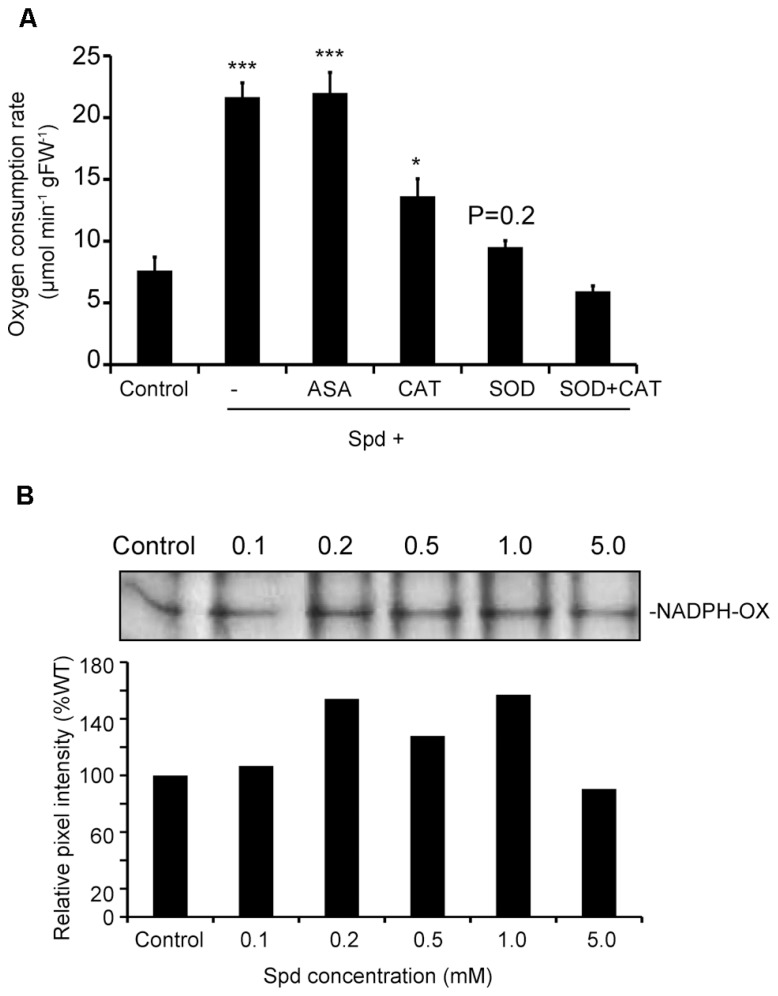
**Combined effect of exogenous Spd and ASA, CAT or SOD on oxygen consumption rate of *Arabidopsis* plants and dose-dependent response of NADPH-oxidase activity by Spd. (A)** Oxygen consumption rate in plants incubated in the respective medium for 10 min. Data are the means of three independent experiments ±SD. Asterisks indicate statistical significant differences (***P < 0.001; *P < 0.05). For Spd+SOD treatment *P* value is indicated. **(B)** Effect of exogenous Spd on NADPH-oxidase activity in Col-0 *Arabidopsis* plants. Plants were treated with 0.1, 0.2, 0.5, 1, and 5 mM Spd for a period of 10 min and relative pixel intensity of lane profile after application of 1 mM Spd was assessed.

To prove the link between the produced ROS and increased respiration, we tested whether quenching of ROS would alleviate the effect of Spd on respiration. Spd was added in combination with ASA, a scavenger of $O_{2}^{•-}$ and H_2_O_2_, catalase (CAT), a scavenger of H_2_O_2_ and SOD, a scavenger of $O_{2}^{•-}$ . Addition of ASA to the medium failed to produce a significant effect on Spd-induced respiration, whereas CAT led to a 37% decrease over the rate found in Spd treatments (**Figure 3A**). The addition of SOD to the Spd- containing medium alleviated the effect of Spd to a greater extent leading to an overall reduction of 57% over the Spd treated plants, to a rate similar to that determined for the untreated plants (control). Finally, treatment with both, SOD and CAT in addition to Spd further reduced the respiration rate, rendering it lower than that determined in the untreated plants. These results suggest that PA-dependent ROS and particularly $O_{2}^{•-}$ are required for induction of the increased oxygen consumption rate.

Previous work from our laboratory established the role of PAs and NADPH-oxidase in the developmental fate of isolated protoplasts (Papadakis and Roubelakis-Angelakis, 2005). Considering that Spd led to a more significant increase of $O_{2}^{•-}$ , than of H_2_O_2_ we hypothesized that $O_{2}^{•-}$ is produced *via* the activation of the NADPH- oxidase. Indeed simultaneous treatment of Col-0 *Arabidopsis* plants with Spd and the NADPH-oxidase blocker DPI led to a significant reduction in both H_2_O_2_ and $O_{2}^{•-}$ in the treated plants (**Figures 2A,B**), providing strong evidence for the participation of the NADPH-oxidase in the Spd-induced ROS accumulation.

So far there are strong indications that there is interplay between Spd and NADPH- oxidase in the generation of ROS which induces enhancement of respiration in *Arabidopsis*. To further verify this result we studied the effect of Spd as a dose-response on NADPH-oxidase activity in Col-0 *Arabidopsis* plants. Treatment with a range of Spd concentrations resulted to a dose-dependent effect in the increase of NADPH-oxidase activity up to a saturation point, and decreased thereafter as indicated by an in gel enzymatic assay (**Figure 3B**). Lower concentrations of Spd (0.1, 0.2, and 1 mM) increased NADPH-oxidase activity compared with the control plants, whereas higher concentration (5 mM and above) decreased NADPH-oxidase activity. These results suggest that low concentrations of Spd can induce NADPH-oxidase.

### AtPAO3 REGULATES THE BALANCE BETWEEN H_2_O_2_ AND $O_{2}^{•-}$

**FIGURE 4:**
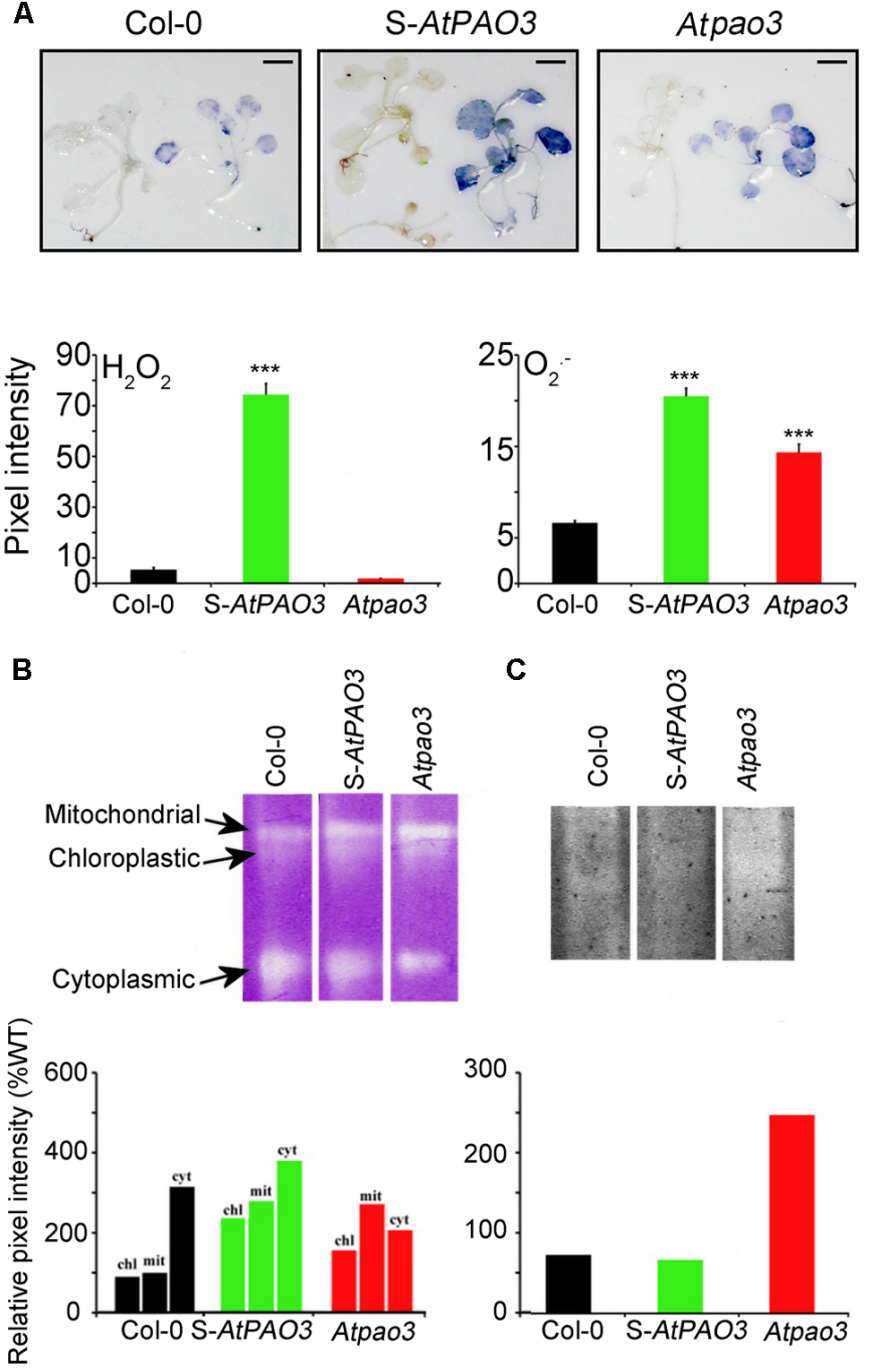
***In situ* ROS in WT, S-*AtPAO3,* and *Atpao3 Arabidopsis* plants. (A)** *In situ* ROS detection in WT, S-*AtPAO3* and *Atpao3* plants. Data are from a single representative experiment, repeated three times, and densitometric analysis. Data are the means of three different positions on a leave. Asterisks indicate statistical significant differences from the Col-0 (***P < 0.001). Scale bars, 2.8 cm. **(B)** Native electrophoresis and activity staining of SOD and densitometric analysis of isoenzymes. **(C)** Native electrophoresis and activity staining of APX and densitometric analysis.

Recent data have shed light on the biochemical role of plant intracellular PAOs, which interconvert Spm to Spd and Spd to Put, reversing the PA biosynthetic pathway (Fincato et al., 2011; Ahou et al., 2014). We have previously shown that plants overexpressing *AtPAO3* efficiently oxidize Spd to Put producing H_2_O_2_ (Moschou et al., 2008c; Wu et al., 2010; Fincato et al., 2011). S-*AtPAO3* plants overexpressing the peroxisomal *AtPAO3* and loss-of-function *Atpao3* seem to be valuable tools in the study of PA-induced respiration in *Arabidopsis* due to the localization of AtPAO3 in the peroxisomes which are in proximity to mitochondria. Indeed, *in situ* detection of ROS in the S-*AtPAO3 Arabidopsis* plants showed that they exhibited a higher but balanced production of H_2_O_2_ and $O_{2}^{•-}$ compared with Col-0 plants (**Figure 4A**). In contrast, *Atpao3* plants accumulated significantly lower levels of H_2_O_2_ when compared with Col-0 plants, but increased levels of $O_{2}^{•-}$ . Therefore, S-*AtPAO3* and *Atpao3* plants are good models for studying the differential effects of H_2_O_2_ and $O_{2}^{•-}$ in oxygen consumption.

Next, we examined a possible contribution of the antioxidant machinery in the observed differences in ROS levels in S-*AtPAO3* and *Atpao3* plants. We determined the contribution of SOD and APX, a well-established H_2_O_2_ scavenger, in the observed differences, between Col-0, S-*AtPAO3* and *Atpao3* in H_2_O_2_ and $O_{2}^{•-}$ levels. Accumulation of $O_{2}^{•-}$ in S-*AtPAO3* and *Atpao3* plants coincided with increased SOD activity in mitochondria and chloroplasts (**Figure 4B**). In contrast, APX was elevated in the *Atpao3*, but reduced in S-*AtPAO3* plants (**Figure 4C**). APX has a high affinity for H_2_O_2_ and therefore is downregulated at higher levels of H_2_O_2_ (Asada, 1992), which perhaps can explain the decrease of APX observed in S-*AtPAO3* plants, with increased H_2_O_2_ levels. These results suggest that at least APX and SOD mirror the changes of ROS levels observed in S-*AtPAO3* and *Atpao3*.

### DEREGULATION OF AtPAO3 RESULTS TO CHANGES IN OXYGEN CONSUMPTION RATE

**FIGURE 5:**
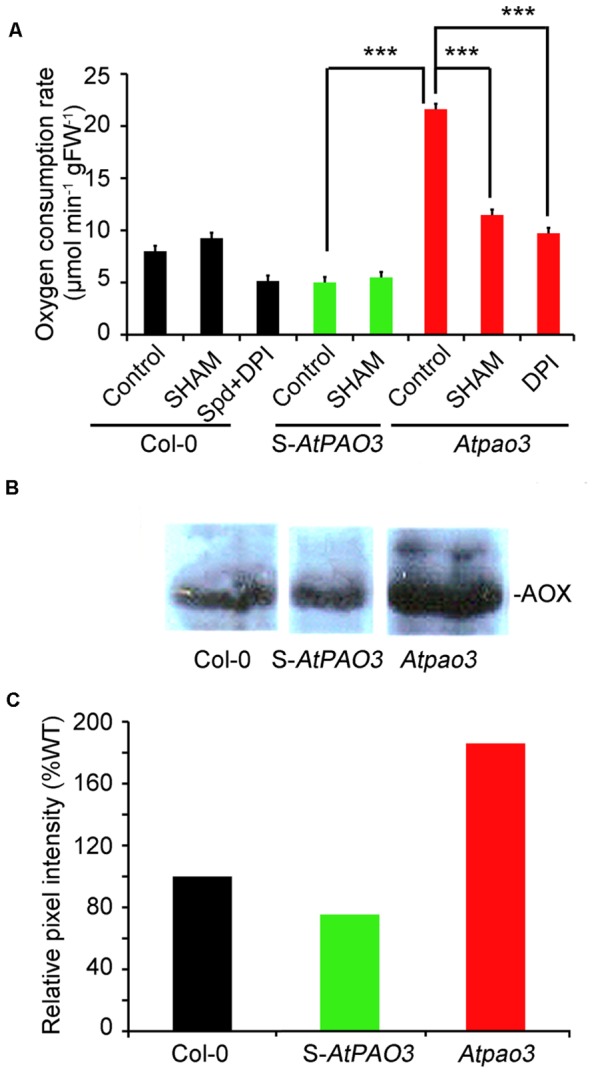
**Oxygen consumption rate in Col-0, S-*AtPAO3* and *Atpao3 Arabidopsis* plants. (A)** Oxygen consumption rate in Col-0, S-*AtPAO3* and *Atpao3* plants. Rate of oxygen consumption was determined in untreated plants and plants treated with the AOX blocker SHAM, exogenous Spd and DPI. Data are the means of three different positions on a leave. Asterisks indicate statistical significant differences from the S-*AtPAO3* control (***P < 0.001). **(B)** Western blot of AOX immunoreactive protein levels in Col-0, S-*AtPAO3* and *Atpao3* plants. Data are from a single representative experiment, repeated three times. **(C)** Densitometric analysis of AOX immunoreactive protein in **(B)**. Data are from a single representative experiment, repeated three times.

Overall the data presented so far support the role of Spd as an inducer of mitochondrial respiration *via* the NADPH-oxidase generated $O_{2}^{•-}$ in Col-0 plants. This response is alleviated mostly by the action of the NADPH-oxidase blocker, DPI. Furthermore, loss-of-function mutant plants for the peroxisomal *AtPAO3* gene accumulate $O_{2}^{•-}$ , but not H_2_O_2_ in contrast to S-*AtPAO3* overexpressing plants, which accumulate both $O_{2}^{•-}$ and H_2_O_2_. Considering the above, we determined the oxygen consumption rate in the S-*AtPAO3* transgenics and the *Atpao3* mutants to test whether the differential accumulation of ROS in the two genotypes leads to altered oxygen consumption. Indeed, the three tested genotypes exhibited notable differences in their capacity to consume oxygen (**Figure 5A**). The *Atpao3* plants exhibited the highest rate of oxygen consumption among the tested plants, showing a 2.7-fold increase over the Col-0 plants and a 4.3-fold increase over the S-*AtPAO3* plants. Interestingly, the increase in oxygen consumption of *Atpao3* plants resembled the effect of exogenous Spd in Col-0 plants.

Next, we examined whether the increase in oxygen consumption of *Atpao3* plants is $O_{2}^{•-}$ -dependent. To test this, we treated *Atpao3* with DPI, and as a control, we treated Col-0 plants simultaneously with Spd and DPI (**Figure 5A**). Treatment of *Atpao3* plants with DPI reduced the respiratory activity to the control levels, suggesting that increase of oxygen consumption in *Atpao3* plants depends on the production of $O_{2}^{•-}$ by NADPH-oxidase. Similarly, Spd plus DPI treated Col-0 plants showed similar oxygen consumption rate to the untreated Col-0 plants, producing an effect equivalent to the DPI-induced decrease in the $O_{2}^{•-}$ accumulation reported earlier.

We hypothesized that the increase observed in the *Atpao3* plants could be due to an increased contribution of the AOX pathway. To test this, we used the AOX pathway blocker SHAM. Indeed, application of SHAM to the *Atpao3* plants exerted a dramatic decrease in the oxygen consumption rate, suggesting the participation of the AOX pathway in the $O_{2}^{•-}$ -induced oxygen consumption (**Figure 5A**). In addition, the levels of the immunoreactive AOX protein in *Atpao3* were significantly higher compared to the rest of the tested plants (**Figures 5B,C**). These results suggest that the $O_{2}^{•-}$ -dependent increase of oxygen consumption in *Atpao3* plants is exerted through the AOX pathway.

## DISCUSSION

Exogenous Spm application to tobacco plants leads to mitochondrial dysfunction and to transcriptional activation of the AOX pathway, while small molecular weight antioxidants efficiently attenuate this induction, suggesting a possible involvement of ROS in this Spm-signaling pathway (Takahashi et al., 2003). A long standing notion proposed that PA oxidation results in induction of signaling cascades through H_2_O_2_, since H_2_O_2_ is a direct product of PA oxidation (Moschou et al., 2008b). However, recent evidence suggests that other ROS types may as well contribute to PA-dependent signaling cascades and that the PAs-ROS crosstalk stretches beyond H_2_O_2_ (Zepeda-Jazo et al., 2011; Velarde-Buendia et al., 2012).

In this work, application of exogenous PAs to Col-0 *Arabidopsis* plants stimulated the oxygen consumption rate; the stronger effect was exerted by Spd. Exogenous application of Spd is expected to increase H_2_O_2_ content through the PA oxidation/backconvertion pathway (Yoda et al., 2003; Moschou et al., 2008b). Therefore, we hypothesized that this product could be responsible for the increase in oxygen consumption. Surprisingly, H_2_O_2_ scavenging did not significantly attenuate the PA-dependent oxygen increase. On the other hand, exogenous SOD or DPI ameliorated the oxygen increase, caused by the exogenous Spd application. This denotes that the increase in oxygen consumption relies on $O_{2}^{•-}$ production, and suggests that exogenous PA induces $O_{2}^{•-}$ production by NADPH-oxidase intriguingly, DPI exerts a stronger effect than SOD. This may be due to the fact that SOD is expected to scavenge intercellular $O_{2}^{•-}$ produced by NADPH-oxidase, giving rise to H_2_O_2_, while DPI is a plasma membrane permeable suicidal NADPH-oxidase inhibitor, that could efficiently prevent production of higher amount of $O_{2}^{•-}$ . Surprisingly, CAT mimicked the DPI effect. Nevertheless, it should be noted that recent evidence suggests that CAT participates in the induction of cell death (Hackenberg et al., 2013), therefore perplexing the interpretation of the data obtained with the use of CAT. ASA, on the other hand, did not alleviate oxygen consumption increase. It should be noted that ASA is a reducing agent, thereby directly affecting the ETC, and scavenges both $O_{2}^{•-}$ and H_2_O_2_ (Foyer and Noctor, 2011). These results suggest that the PA-derived oxygen consumption increase depends mostly on $O_{2}^{•-}$ production by the PA-induced NADPH-oxidase.

Exogenous PA at relatively high concentrations stimulated production of $O_{2}^{•-}$ in human neutrophils (Guarnieri et al., 1987). In our study, exogenous application of Spd in *Arabidopsis* increased the content of H_2_O_2_ produced through PAO, as previously suggested for other plant species (Yoda et al., 2006; Wu et al., 2010; Moschou and Roubelakis-Angelakis, 2011; Tisi et al., 2011). Nevertheless, exogenous Spd induced a significant increase in the levels of $O_{2}^{•-}$ along with an increase in NADPH-oxidase activity. Interestingly, previous studies suggested that Spd induces autophagy (self-consumption) in non-plant models and is an important surveillance mechanism that rather restricts ROS production (Eisenberg et al., 2009). In accordance, during protoplast isolation from tobacco (Papadakis and Roubelakis-Angelakis, 2005), induction of NADPH-oxidase and the concomitant production of $O_{2}^{•-}$ was highly suppressed by PAs. This discrepancy between our current and previous work could be due to the fact that in Papadakis and Roubelakis-Angelakis paper, PAs were added to the protoplasts and then NADPH-oxidase was purified and assayed. On the contrary, in this study PAs were added exogenously and NADPH-oxidase assessment was performed by an in-gel assay omitting further purification steps. This allows us to hypothesize that exogenous PAs may antagonize for Ca^2^^+^in NADPH-oxidase preparations. Calcium is required for NADPH-oxidase activation. Therefore, we assume that NADPH-oxidase cannot accommodate Ca^2^^+^ in the presence of PAs on its binding. In addition, in the previous study $O_{2}^{•-}$ content was determined by a chemiluminescence assay, while in this work we used an *in situ* detection protocol, which seems to be a more accurate and reliable method (Song et al., 2006). Therefore, PAs and especially Spd seem to positively affect NADPH-oxidase *in planta*, unlike in *in vitro* systems. In addition, a species-related differential effect on NADPH-oxidase of PA cannot be ruled out (tobacco versus *Arabidopsis*).

From the aforementioned, it is conceivable that production of $O_{2}^{•-}$ may depend on a PAO system. There are two main PA oxidation regimes: an apoplastic and an intracellular one. Several plant species, especially monocots, possess the ability to oxidize higher PAs in their apoplastic compartments. The oxidative reaction is executed by DAOs and PAOs residing in this compartment. However, the former enzymes show low affinity for higher PAs such as Spd. Notably, apoplastic PAOs in *Arabidopsis* are missing. There are five genes encoding for PAOs in *Arabidopsis.* The inducible *AtPAO1* (Tavladoraki et al., 2006) and constitutively expressed *AtPAO5* (Ahou et al., 2014) encode for cytoplasmic enzymes, oxidizing Spm, while *AtPAO2, 3, 4* encode peroxisomal proteins (Kamada-Nobusada et al., 2008; Moschou et al., 2008c), oxidizing Spd (AtPAO2, 3) and Spm (AtPAO2,3,4). On the other hand, in *Arabidopsis* there are ten genes encoding DAOs, four of which have been characterized. One of them is an apoplastic enzyme; however, six remain to be characterized (Moschou et al., 2013; Planas-Portell et al., 2013). These suggest that most likely the contribution of the apoplast to the Spd-dependent production of $O_{2}^{•-}$ is rather minimal in *Arabidopsis*.

To further examine whether intracellular PAOs are responsible for inducing production of $O_{2}^{•-}$ , we employed genetic means. The best characterized so far enzymatic activity that oxidizes mostly Spd and to a smaller extent Spm in *Arabidopsis* is that of the peroxisomal AtPAO3 (Moschou et al., 2008c; Fincato et al., 2012). In mammals and plants, PA oxidation has been implicated in the execution of PCD (Yoda et al., 2003; Tavladoraki et al., 2006; Moschou and Roubelakis-Angelakis, 2013). It has been exemplified that there is a direct relationship between PCD and the levels of cytotoxic PA catabolic products, i.e., H_2_O_2_ and aminoaldehydes. For example, during *Helicobacter pylori* infection that contributes to gastric cancer, PA-derived H_2_O_2_ coincides with PCD induction (Chaturvedi et al., 2004). However, PA-derived H_2_O_2_ seems to be a double-edged sword since oxidation by SMO could perhaps contribute to the eradication of tumor cells (Babbar et al., 2007). In tobacco, overexpression of apoplastic PAO is accompanied by premature cell death of xylem tissue (Tisi et al., 2011). Interestingly, exogenous supply of Spd to maize root tips highly expressing *PAO* alters cell cycle distribution, toward quiescence and induces PCD (Tisi et al., 2011). In addition, premature cell death of xylem hinders the proper differentiation of the secondary cell wall, which is normally deposited before PCD induction in xylem. Importantly, a H_2_O_2_ scavenger partially ameliorates Spd-induced effects. In addition, 4-aminobutanal which is an additional oxidation product of Spd, failed to mimic Spd effects, indicating that PAO-derived H_2_O_2_ is sufficient to induce PCD independently of aminoaldehydes.

In contrast to the previous, the PA backconversion pathway seems to have completely distinct functions, which remain largely elusive. It was shown that AtPAO3 is an important component of pollen tube elongation (Wu et al., 2010). More specifically, AtPAO3 generates H_2_O_2_ which positively affects the permeability of a plasma membrane-residing Ca^2^^+^influx channel. As a result, the intracellular concentration of Ca^2^^+^ increases, thereby promoting pollen tube elongation. In loss-of-function *Atpao3* reduction of pollen tube elongation, and in a physiological context reduced fertility was evident. In addition, a role for the PA backconversion pathway was hypothesized with respect to dehydration response of *Arabidopsis* (Alcazar et al., 2011a). The putative paralog of *AtPAO3* gene, *AtPAO2* is upregulated by drought stress in a similar fashion as *RD29A* and *RD22*.

A number of PAOs have been implicated in the PA backconversion pathway in *Arabidopsis* and, unlike in mammals, plant PAOs did not require acetylated derivatives (Moschou et al., 2008c). We observed that plants overexpressing *AtPAO3* showed increased content of H_2_O_2_ consistent with its role in PAs oxidation. Surprisingly, this H_2_O_2_ production led to a significant $O_{2}^{•-}$ increase, while *Atpao3* mutants showed reduced levels of H_2_O_2_ but increased $O_{2}^{•-}$ . This implies that loss of AtPAO3 caused an increment of $O_{2}^{•-}$ *versus* H_2_O_2_. In animal cells, ROS have also been shown to play an important role in maintaining the balance between cell proliferation and differentiation. A redox-dependent signaling pathway controls the induction of cell division through the regulation of *cyclinD1* expression (Burch and Heintz, 2005). Distribution of specific ROS appears to act as an important signal at the transcriptional and posttranscriptional levels during cell-cycle progression (Menon and Goswami, 2007). In *Drosophila*, changing ROS balance can switch the status of hematopoietic cells from proliferation to differentiation (Owusu-Ansah and Banerjee, 2009). In *Arabidopsis*, it was shown that $O_{2}^{•-}$ accumulates primarily in the root meristematic zone, whereas H_2_O_2_ accumulates mainly in the elongation zone (Tsukagoshi et al., 2010). Moreover, it has been shown that Mn-SOD activity regulates cell-cycle progression through modulation of ROS levels, which control expression of both the *cyclinB1* and *cyclinD1* genes in mouse cells (Sarsour et al., 2008). The authors proposed that $O_{2}^{•-}$ regulates the proliferative cycle, whereas H_2_O_2_ induces quiescence and differentiation. Therefore, in the root elongation zone, the ratio between $O_{2}^{•-}$ and H_2_O_2_ is decreased (Tsukagoshi et al., 2010).

In our study, AtPAO3 was shown to be an important factor for balancing $O_{2}^{•-}$ and H_2_O_2_. Increased levels of $O_{2}^{•-}$ *versus* H_2_O_2_ were detected in the absence of AtPAO3, perhaps due to the increased activity of APX, which scavenges H_2_O_2_. S-*AtPAO3* plants show reduced expression of APX but increased expression of mitochondrial and chloroplastic SOD isoenzymes, while *Atpao3* show significantly increased expression of APX, mitochondrial and chloroplastic SOD. Noteworthy, the increased isoenzymes are in proximity to peroxisomes. These changes are in accordance with the ROS levels detected in these plants. However, further studies are required to elucidate whether the increased/decreased expression of these antioxidants controls ROS levels or alternatively, whether ROS levels control the induction/reduction of these genes/enzymes. Although this may sound like a “chicken or the egg” question it merits careful examination to further understand the regulation of ROS homeostasis. We can speculate that similar regulation of the $O_{2}^{•-}$ /H_2_O_2_ ratio takes place during other developmental transitions, apart the ones reported in the root (Tsukagoshi et al., 2010) like for example during pollen tube growth, which could contribute to the failure of *Atpao3* pollen tube elongation (Wu et al., 2010).

Overexpression of *SSAT* in mice, an acetylase required to direct PAs in non-plant models toward the PAO pathway, leads to increased H_2_O_2_ and carbonyl content, and reduced SOD, CAT, and cyt CYP450 2E1 expression, responsible for xenobiotic metabolism. This suggests that transgenic mice are hypersensitive to stress, leading to cell death, and they also are sluggish and less hostile (Kaasinen et al., 2004). Interestingly, although S-*AtPAO3* plants accumulate significantly higher amounts of ROS, they do not show symptoms of chronic stress. Tobacco plants overexpressing apoplastic PAO exhibit increased SOD and CAT expression, which do not exert a protective effect, but rather this increased expression represents an attempt to scavenge surplus H_2_O_2_ produced by continuous PA oxidation. The previous suggests that as in animals, constitutive apoplastic PA oxidation in plants can lead to chronic oxidative stress (Moschou et al., 2008a).

On the contrary, the AtPAO3 backconversion pathway seems to have a completely different function. We show that Spd oxidation by AtPAO3 is required for a balanced respiration through the cyt-c and AOX pathways. Notably, overproduction of PA-derived H_2_O_2_ in the S-*AtPAO3* plants results in a small decrease of oxygen rate consumption, but not in induction of the AOX pathway. To the contrary, *Atpao3* plants show increased oxygen consumption through the AOX pathway. Interestingly, this increase is attenuated by application of DPI, which specifically blocks $O_{2}^{•-}$ generation by NADPH-oxidase. It was reported that a microtubule associated kinesin and a mitochondrial channel are able to regulate the balance between cyt-c and AOX pathways (Yang et al., 2011). In addition, it has been hypothesized that the ratio of (singlet + $O_{2}^{•-}$ )/H_2_O_2_ determines PCD initiation during stress (Sabater and Martin, 2013). The previous allow us to propose that an increased ratio of $O_{2}^{•-}$ /H_2_O_2_ leads to increased oxygen consumption through the AOX pathway. Likewise, it has been reported that $O_{2}^{•-}$ is sufficient to induce *AOX1a/b* genes in rice (Li et al., 2013). These results demonstrate that depletion of AtPAO3 leads to higher production of $O_{2}^{•-}$ , which in turn activates the AOX pathway.

In conclusion, our results allow us to propose that AtPAO3 is required for balancing $O_{2}^{•-}$ /H_2_O_2_ production. An imbalance of the $O_{2}^{•-}$ *versus* H_2_O_2_ production leads to activation of AOX pathway and increases oxygen consumption. The next critical step to advance our understanding on the role of PA backconversion, and its interplay and crosstalk with ROS will be the genetic dissection of PA backconverting pathways, and their molecular effectors.

## Conflict of Interest Statement

The authors declare that the research was conducted in the absence of any commercial or financial relationships that could be construed as a potential conflict of interest.

## Footnotes

1 http://rsb.info.nih.gov/ij/index.html

2 http://www.spss.com

3 http://www.jmp.com
